# Effects of weather scenarios and fertilizer on maize growth and yield: Insights from a greenhouse experiment

**DOI:** 10.1371/journal.pone.0318121

**Published:** 2025-03-03

**Authors:** Souand P G Tahi, Kolawolé Valère Salako, Vinasetan Ratheil Houndji, Romain Glèlè Kakaï

**Affiliations:** 1 Laboratoire de Biomathématiques et d’Estimations Forestières, Faculty of Agronomic Sciences, University of Abomey-Calavi, Cotonou, Benin; 2 Institut de Formation et de Recherche en Informatique, University of Abomey-Calavi, Cotonou, Benin; National Museums of Kenya, KENYA

## Abstract

Maize is a major crop for food security, but its cultivation is threatened by climate change. Climate may affect the response of maize to fertilizer. This study examined the impact of weather parameters in combination with fertilizer types on maize growth and yield parameters in Benin. The experiment involved two sets of climatic scenarios. Scenario 1 (weather 1) had a moderate range of minimum and maximum temperatures and maximum humidity suitable for maize cultivation in Benin. Scenario 2 (Weather 2) featured a broader range of parameter values below and above those of Weather 1. Five types of fertilizers were tested: Organic (Cow dung), Chemical (NPK), Intermediate 1 (mixture of high NPK and low Cow dung), Intermediate 2 (mixture of middle NPK and middle cow dung), and Intermediate 3 (mixture of high cow dung and low NPK). These factors were combined in a split-plot design and data were collected on maize germination, growth, and yield variables. Models such as DNNsurv, Cox, linear mixed effect, and decision trees were used for data analysis. Results revealed that maize seeds had a higher probability of germination between 2 to 5 days after sowing, with over 80% of the seeds germinating the fifth day. Intermediate 1 and organic fertilizers were particularly effective in promoting maize growth, resulting in larger diameters and heights. Organic, chemical, and intermediate 1 fertilizers led to higher yields under weather scenario 2, while intermediate 3 and organic led to higher yields under weather 1, suggesting that organic fertilizers could be more sustainable and cost-effective than mineral fertilizers. Additionally, Weather 2 was associated with higher maize yields suggesting that, a relatively broader range of climate parameters would positively affect maize yield. These findings can assist farmers and policymakers in making well-informed decisions regarding the most suitable fertilizers to use under various weather conditions, maximizing their yield and profits.

## Introduction

Maize (*Zea mays*) is one of the most important cereal crops for human and animal feed [1]. It provides 44% of the world’s cereal intake [2]. Its global production area has doubled since 1961, from 106 million to 197 million hectares, with an expansion reported in the 2000s [3]. Similarly, Maize productivity has increased in developed countries from 2 tonnes/ha to 5.8 tons/ha [4]. Based on FAO statistics, its annual average consumption is 18.5 kg per capita, with Africa recording the highest rates of consumption, outstripping the rate for livestock (30.46 kg/capita/year) [3,5]. In addition to its use for food, maize is also used for bioethanol production [6].

In sub-Saharan Africa, maize is also the main cereal crop, covering 40 million hectares, with a daily average of 100g per capita [7]. According to [8], more land in this region is being used for maize production. Its annual production is estimated at 25.98 million tonnes in 2020, with Nigeria, Mali, Ghana, Burkina Faso, and Benin accounting for 85.13% of total production [9]. Over the decades, maize yield expansion in cultivable sub-Saharan Africa has been linked to expanding arable land, except in the Central African Republic, Côte d’Ivoire, Eswatini, Ethiopia, Lesotho, Mauritius, Somalia, South Africa, and Zambia [7]. Despite maize’s importance in the fight against food insecurity, its production faces various problems among which are fertilization, soil degradation, climate change, etc.

Crop production is primarily influenced by various biotic and abiotic factors, and increasingly threatened by climate change. Indeed, climate change imposes new challenges in many fields, including agriculture. Extreme weather conditions can significantly impact crop yield through biotic stress, such as disease and insect infestation [10], or abiotic stress, such as drought, flooding, thermal stress, and soil salinity. Forecasted consequences of climate change have shown that rising temperatures and decreased precipitation could result in poorer yields if adaptation measures are not taken. Other studies have also highlighted the anticipated negative impacts of climate change on maize yield ‘[11]. Therefore, it is essential to explore new climate-resilient cropping strategies, specifically adapted to the region’s changing and extreme climatic conditions. Sub-Saharan Africa is very susceptible to climate change [12,13]. In this zone, crop production depends heavily on precipitation, and off-season cultivation is not well-developed. Its production season usually runs from May to September, with annual rainfall ranging from 200 mm to 1200 mm.

In addition to climatic challenges, soil health, and fertilization are significant issues in maize production. Conventional agriculture has become heavily dependent on fertilizers, particularly chemical fertilizers. However, chemical fertilizers have severe implications for human and soil health. Continuous use of this fertilizer causes physicochemical and biological alteration of the soil, leaching of nutrients, and contamination of groundwater [14], and Benin is not immune to these challenges. The challenges posed by climate change and soil degradation in Benin emphasize the urgent need for research to enhance maize yield under these pressures. With most of Benin’s arable land already degraded due to intensive farming and unsustainable practices, such as the overuse of chemical fertilizers, climate variability further exacerbates these issues. Changes in rainfall patterns, increased temperatures, and prolonged dry spells reduce soil fertility and crop productivity, making sustainable agricultural solutions a priority. Since maize is more vulnerable to climate change than other cereals (e.g., sorghum or millet), some experts advise improving fertilizer doses for crops experiencing rising temperatures due to climate change [15]. Sub-Saharan Africa’s population is expected to double by 2030 [16]; hence, increasing productivity is necessary to ensure regional food security without compromising soil health. Adopting sustainable agricultural practices and finding solutions to climate challenges have become priorities for sustainable agriculture in the region. Several studies have compared the effects of different types of fertilizer on maize yield in sub-Saharan Africa [17,18]. In addition, various studies assessed the impacts of climatic parameters on maize yield [19–21].

This study evaluated how fertilizer type and weather association parameters affected maize growth and yield in Benin. To achieve this, we set up an experiment in a greenhouse and i) determined the germination time as a function of fertilizer and weather association parameters, ii) assessed the influence of fertilizers and weather associations on growth parameters, and iii) evaluated the variation in maize yield in response to fertilizer and weather associations.

## Materials and methods

### Experimental design

An experiment was carried out in a 150 *m*^2^ greenhouse in Sekou, Benin (6°37’39.9"N 2°13’51.9"E) to evaluate the combined effects of weather scenarios and fertilizer types on maize yield. Weather scenarios were defined by specific combinations of weather parameters (minimum and maximum température and maximum humidity), each characterized by precise value ranges. The study was performed in two distinct phases: from January 19 to April 17, 2023 (phase 1), and from May 6 to August 2, 2023 (phase 2). Each phase evaluated the effect of one weather scenario and fertilizer types on maize parameters. For example, phase 1 assessed the impact of weather scenario 1 and fertilizer types on maize productivity. The experimental design was a split-plot design, with weather scenarios as the primary factor and fertilizer levels as the secondary factor (see the Supporting information, S1 Fig). Fertilizer type was randomized within the block, and the process was repeated thrice to ensure dependable results and limit random errors. Two weather scenarios were considered; scenario 1 (weather 1) with moderate range of minimum and maximum temperatures and maximum humidity and scenario 2 (Weather 2) with a broader range of parameter values below and above those of Weather 1. According to Tahi et al. [22], these weather patterns are ideal for the optimal production of maize in Benin. Additionally, five types of fertilizers were tested: Organic (Cow dung), Chemical (NPK), Intermediate 1 (mixture of high NPK and low Cow dung), Intermediate 2 (mixture of middle NPK and middle cow dung), and Intermediate 3 (mixture of high cow dung and low NPK). During the experiment, the plants received two daily hand irrigations (7 a.m. and 4:30 p.m.) of the standard water quantity. Tmin, Tmax, and Umax were measured using temperature and humidity sensors (*Thermometer Type: Digital, Thermometer Use: Bath Thermometers, Place of Origin: Guangdong, China, Type: Household Thermometers, Product Type: Temperature Humidity Meters, Material: plastic, Brand Name: ThermoPro*).

After weeding, 31,250 plants are recommended per hectare for maize production. According to [23] recommendations, 50 kg of urea and 100 to 200 kg of NPK (nitrogen, phosphorus, and potassium) are required per hectare for chemical fertilization. This study applied the recommended proportion per plant, considering 150 kg of NPK and 50 kg of urea per hectare (Table 1). Regarding organic fertilizers, the authors suggested 20 to 50 tons per hectare. Dried cow dung was used in this study. Based on 20 tonnes per hectare, the estimated quantity per plant was applied. Each block consists of a set of 30 observation units, each representing an experimental unit. The maize variety used was TZEE W pop QPM, with high-quality seed supplied by the National Institute for Agronomic Research of Benin (INRAB).

**Table 1:** Weather scenarios and fertilizer levels used in the greenhouse experiment. Values for the weather variables are ranges. Tmin = minimum temperature, Tmax = maximum temperature, Umax = Maximum humidity.

Variables	Modalities	Values
Weather scenarios		
Weather 1	Tmin (° C)	22.1 - 23.9
	Tmax (° C)	29.8 - 33.6
	Umax (° C)	93.5 - 96.3
Weather 2	Tmin (° C)	21.3 - 24
	Tmax (° C)	29.4 - 35.9
	Umax (%)	87.3 - 102.3
Fertilizer (g)		
Organic	Dried cow dung	640
Chemical	NPK	480
	Urea	1.6
Intermediate 1	1/4 Dried cow dung	160
	3/4 NPK	360
	3/4 Urea	1.2
Intermediate 2	1/2 Dried cow dung	320
	1/2 NPK	240
	1/2 Urea	0.8
Intermediate 3	3/4 Dried cow dung	480
	1/4 NPK	120
	1/4 Urea	0.4

The greenhouse design was carefully planned, considering aspects such as wind direction, soil configuration, and site characteristics. The microclimate was rigorously controlled by adjusting the shade cloth, basins of water in the four corners of the greenhouse, and ventilation systems under constant supervision.

### Data collection

Germination, growth, and yield parameters were recorded for each of the 450 plants in each experiment phase. Germination time was recorded over ten days from planting time. Ungerminated seeds by the tenth day were replanted. One week after germination, growth parameters were recorded weekly during six weeks. These include height (in cm), stem diameter (in cm), number of leaves per plant, length (in cm), and width of tallest leaf (in cm). Growth data collection spanned over six weeks, mirroring the crucial growth cycle of the variety under study. This variety reaches flowering on approximately the $35^{th}$ day, marking a significant milestone in its total cycle of 80 days [24]. Most of these parameters were meticulously collected using a measuring tape (*Length: 2M, 1.5 m, Material: plastic, Blade Width: 0, Blade Thickness: 0, Measurement system: Metric qraduation*). Yield parameters were measured eight days after the end of the cycle ( 88 days). These included the number of cobs per plant, the fresh weight of maize cobs in spathe (in g), the length and width of the cobs (in cm), the fresh weight (in g), and the number of seeds. Ten observation units were randomly selected to count the number of grains per maize stalk for each experimental unit. Kernels were then counted on the selected plants. The weight of the maize was measured using an electronic scale (*Place of Origin: Zhejiang, China, Brand Name: beechen, Model Number: SF400, Feature: With Scale Tray, Shape: Rectangle, Maximum Weight Recommendation:10KG, Power Source: Battery*).

### Statistical analysis

#### Germination data.

The germination time of maize kernels was analyzed over ten days. Ungerminated seeds at day ten were considered right-censored (0), while germinated seeds were represented by 1, showing that germination occurred during this period. Two survival models were used and compared to explain maize germination time. These were the Cox proportional hazards model (semi-parametric model) developed by Cox [25] and the Cox-based deep neural network (DNNsurv) (non-parametric model) [26]. Both models evaluate the impact of interest factors (fertilizer and weather association) on germination time. The assumption of proportional hazards was assessed to ensure the constant effect of explanatory variables over time. Thus, 70% of the germination data were used to design the survival model and 30% for testing the performance.

In addition, the non-parametric Kaplan-Meier model (KM) was used to evaluate the probability of germination time [27]. An additional analysis using the log-rank test evaluates the differences in germination between each factor. Metrics used to evaluate Cox proportional and DNNsurv models include the concordance index (C-index), which measures a model’s ability to correctly classify samples according to their germination or non-germination time; the root mean square error (RMSE), which indicates the standard deviation of the residuals; and the mean absolute error (mae), which measures the average of the errors’ magnitude between the predicted and actual values. The mathematical expressions for metrics are shown below.Germination time analysis was performed in Anaconda’s Jupyter 6.3.5.


$$\begin{array}{c} C-index=\frac{\underset{i,j}{\sum }1_{T_{j}<T_{i}}\cdot 1_{\eta_{j}>\eta_{i}}\cdot \delta_{j}}{\underset{i,j}{\sum }1_{T_{j}<T_{i}}\cdot \delta_{j}} \end{array}$$ (1)

$$\begin{array}{c} MAE=\frac{\sum (|y_{i}-\hat{y_{i}}|)}{n}, \end{array}$$ (2)

$$\begin{array}{c} RMSE=\sqrt{MSE}=\sqrt{\frac{1}{n}\overset{n}{\underset{i=1}{\sum }}{\left(y_{i}-{ŷ}_{i}\right)}^{2}}, \end{array}$$ (3)

$\eta_{i}$ is the risk score of unit i, $1_{T_{j}<T_{i}}=1$ if $T_{j}<T_{i}$ else 0; $1_{\eta_{j}>\eta_{i}}$ if $\eta_{j}>\eta_{i}$ else 0. $y_{i}$ is the real variable and $\hat{y_{i}}$ is the estimated variable.

#### Growth and yield parameters.

This analysis focuses on two data types: longitudinal and cross-sectional. Longitudinal data includes weekly growth measurements over 35 days, while cross-sectional data consists of growth parameters collected on the $35^{th}$ day and yield parameters measured on the $88^{th}$ day after sowing.

#### Cross-sectional data.

Cross-sectional data were initially analyzed descriptively. Following this, a two-factor analysis of variance (ANOVA) was performed to examine the effects of weather scenarios and fertilizer types on each growth parameter. A t-test was also conducted to determine whether growth and yield parameters varied under different weather scenarios (See the Supporting information S1 and S2 Tables). After the description, yield parameters were utilized to develop explanatory models using linear and generalized mixed-effect models [28,29]. The linear mixed-effects model was applied to continuous parameters, including cob length, width, maize spathe weight, and maize weight. The generalized mixed-effect models were applied to discrete parameters (i.e. number of seeds and number of cobs). Both models are classified as mixed because they incorporate fixed (i.e. weather association, fertilizer types) and random factors (i.e.block). The models estimate the variance for each fixed and random factors to capture the correlation between observations and model the group effect. This study used two libraries: *’nlme’* [30] and *’glmmML’* [31] to model continuous and discrete maize parameters, respectively. The analysis involved testing the effects of the random factor by using the unconditional mean models. The intra-class correlation coeﬃcient (ICC) was calculated to evaluate its contribution to the total variability. However, interactions between factors were not considered, as most models including these interactions failed to converge. The best variance-covariance matrix structure for residuals was selected by assessing the relevant factors (climatic scenarios and fertilizer types) on yield parameters through a likelihood ratio test. The optimal model was selected to establish the best structure for the random effects matrix. The analysis of variance was performed on models to identify critical factors affecting yield parameters. Given various interest factors, the models’ explicability coeﬃcients were calculated to assess the threshold for the parameters’ explicability. The various analyses of cross-sectional data were performed in R 4.3.1.

#### Longitudinal data.

The longitudinal data analysis involved examining various growth parameters collected weekly, such as height, collar diameter, upper leaf length, width, and the number of leaf. A Pearson correlation matrix was used to highly correlated parameters, applying a threshold of 0.80. The selected parameters were then used to create explanatory models based on linear mixed effects. The input variables for these models included weather scenarios, fertilizer types, blocks, and time. The analysis assessed the impact of the random factor (block) and time using unconditional mean and unconditional growth models. The Intra-class correlation coeﬃcient (ICC) was calculated to assess their contribution to total variability and variability due to time. The interactions between predictors were not considered due to convergence issues in models. However, a likelihood ratio test selected the best variance-covariance matrix structure for residuals. The best model was chosen to select the random effects matrix structure. The analysis of variance underscored the importance of both interest and time factors. The models’ explicability coeﬃcients were determined to assess the parameter’s threshold for explicability across various interest factors. Finally, growth curves were plotted to describe the specific effects of each treatment. All explanatory models based on longitudinal data were carried out in R 4.3.1.

#### Predictive models.

Parallel models derived from decision trees were also applied in this evaluation. These include decision tree (DT) [32], random forest (RF) [33], extreme gradient boosting (XGBoost) [34], gradient boosting regression (GBR) [35], bagging regression (BR) [36], adaptive boosting (AdaBoost) [37], and light gradient boosting machine (light GBM) models [38]. Models were evaluated using metrics such as mean absolute error (MAE), goodness-of-fit coeﬃcient ($R^{2}$), and root mean square error (RMSE). The analyses used the *scikit-learn* library of Anaconda’s Jupyter 6.3.5 environment. The results of these analyses were unsatisfactory (see the Supporting information, S3 Table) and are therefore not detailed.

## Results

### Germination

Maize germination time was analyzed by comparing two modeling approaches: the Cox proportional hazards model and the DNNsurv model. The results revealed that the DNNsurv model outperformed the Cox model, exhibiting low error rates (RMSE = 0.73, MAE = 0.54) and a high concordance index (C-index = 0.54) (Table 2). These findings indicate moderate agreement between the actual and predicted germination times.

**Table 2:** Performance of the Cox and DeepL models based on Root mean square error (RMSE), Mean Absolute Error (MAE) and Coeﬃcient of Agreement (C-index).

Models	RMSE	MAE	C-index
DNNsurv	0.73	0.54	0.54
Cox PH	1.71	1.56	0.45

**Fig 1:**
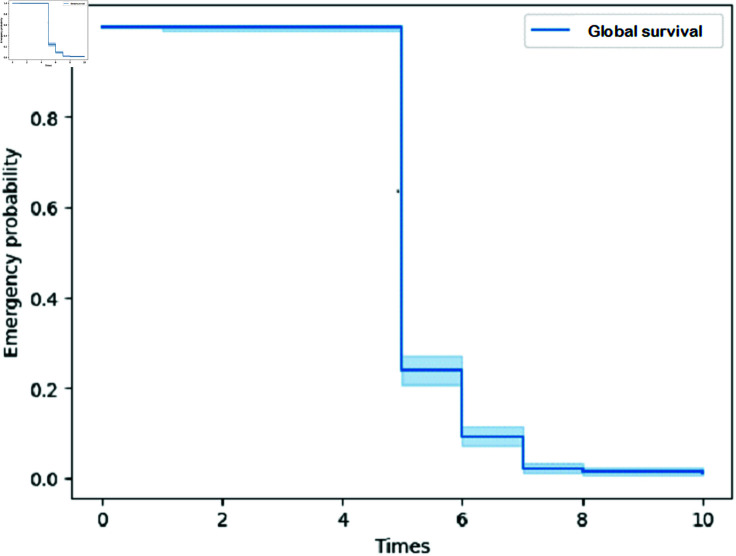
Overall probability of emergence of maize seedlings as a function of time based on the Kaplan-Meier method. The shaded area represents the confidence envelop.

Using the Kaplan-Meier (KM) model, there was no difference in maize germination time based on the types of fertilizer or weather scenarios (see the Supporting information, S2 Fig). Furthermore, the overall survival curve indicated that maize seed started germination on day five and completed on day eight (Fig 1). Moreover, analysis using the log-rank test confirms the findings from the KM model. The probability values from this test for each group of factors were not significant ( fertilizer: log-rank test = 1.79, p-value = 0.180; weather association: log-rank test = 2.37, p-value = 0.12), concluding that germination time was not influenced by fertilizer type or the climate scenarios.

### Growth parameters

**Fig 2:**
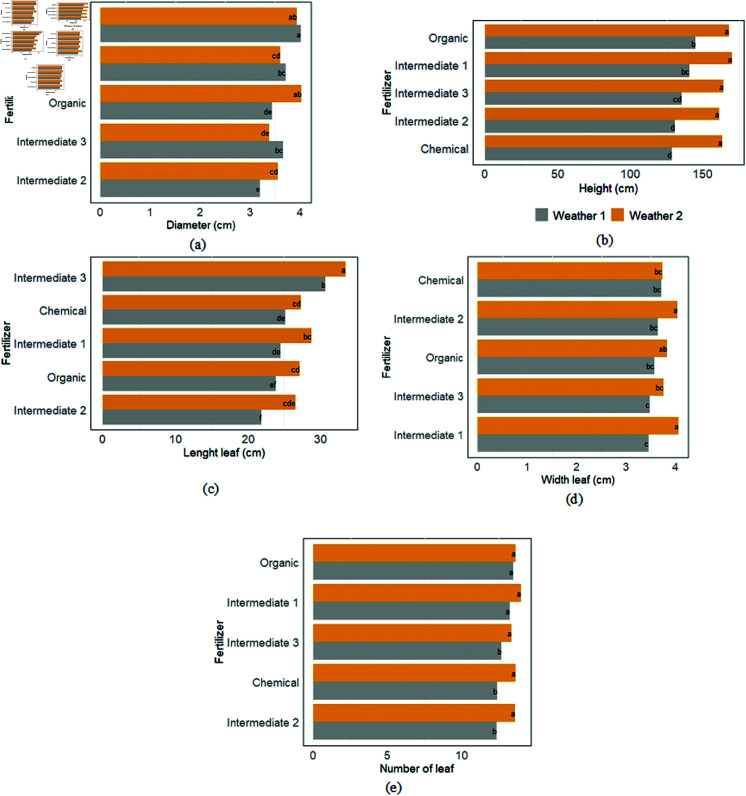
Variation of growth parameters (mean ± standard error) according to fertilizer types and weather scenarios: (a) Diameter in cm, (b) Total height in m, (c) Lenght leaf in cm, (d) width leaf (cm), (e) Number of leaf.

The ANOVA test revealed a significant difference in plant diameter, height, length, and leaf width based on the treatments. However, the number of leaves remained constant across all plants regardless of treatments (Fig 2). Intermediate 1 consistently resulted in larger diameters for weather scenario 1, while intermediate 1 and organic resulted in larger diameters for weather scenario 2 (Fig 2(a)). Concerning the height, the difference was observed only for weather 1, with larger values observed for organic, intermediate 1, and 3 (Fig 2(b)). Furthermore, plants treated with intermediate 3 fertilizer in both weather scenarios exhibited longer leaves (Fig 2(c)). In weather scenario 1, organic, chemical, and intermediate 1 fertilizers resulted in similarly wide leaves. In contrast, intermediate 1 and 2 had the most significant effect in weather scenario 1, particularly in terms of leaf width (Fig 2(d)). The differences in growth parameters based on the weather scenarios were further confirmed through a Student’s t-test analysis (refer to the Supporting information, S2 Table). The highest values for all parameters were recorded under weather scenario 2. Additionally, a Pearson correlation analysis revealed a high correlation coeﬃcient of 0.96 between the “number of leaves” and “height” variables; thus, the “number of leaves” variable was excluded from further analysis.

Linear mixed-effect model analyzed growth parameters according to time, block, fertilizer, and weather conditions.The results indicate that the intra-class correlation coeﬃcient (ICC) for diameter was 2% using the null model. Then, the block has a minor impact on the observed diameter variability, suggesting that the diameter remains consistent across different blocks. However, time variability was estimated at 48%, highlighting a significant impact of time on plant diameter. The best-performing model was the conditional growth model with a first-order autoregressive moving average structure, homogeneous residual variance-covariance matrix, and a variance-component variance-covariance matrix structure. Analysis of variance (ANOVA) on this model demonstrated that time and fertilizer levels significantly influence maize plant diameter, while weather scenarios have no effect (Table 3).

**Table 3:** Effects of weather scenarios and fertilizer types on maïze growth traits: summary of the linear mixed models. ICC = Intra class correation, DF = Degree of Freedom, F = Fisher Statistics, P = P-value, R^2^m = Marginal Rsquare, R^2^C = Conditional R square.

Growth traits	(Intercept)			Time			Fertilizers			Weather scenarios			ICC (bloc) %	ICC (time) %	R^2^m	R^2^C
	*DF*	*F*	*P*	*DF*	*F*	*P*	*DF*	*F*	*P*	*DF*	*F*	* P*				
Diameter	1 (5322)	2631.8	<.0001	1 (5322)	365.6	<.0001	4 (5322)	4.7	0.0009	1 (5322)	0.3	0.6	2	48	0.3	0.3
Height	1(5323)	41.7	<.0001	1 (5323)	1309	<.0001	4 (5323)	10.8	<.0001	1 (5323)	9.4	0.0022	0.6	74.7	0.6	0.7
Width leaf	1 (5287)	921.1	<.0001	1 (5287)	493.9	<.0001	4 (5287)	2.1	0.07	1 (5287)	42.7	<.0001	0.9	28.6	0.3	0.3
Length leaf	1 (4133)	2086.7	<.0001	1 (4133)	420.1	<.0001	4 (4133)	8.7	<.0001	1 (4133)	11.8	6e-04	0.38	9.2	0.1	0.1

Regarding height, the ICC of the null model was 0.68%, suggesting minimal variability associated with the block. Additionally, time variability was estimated at 75%, underlining the importance of time in plant height variation. The conditional growth model was also identified as optimal with a first-order autoregressive moving average structure, homogeneous residual variance-covariance matrix, and a variance-component variance-covariance matrix structure. ANOVA analysis of the height model revealed significant effects of time, fertilizers, and weather conditions on maize plant height (Table 3).

With leaf width, the ICC was 0.91%, suggesting little impact from different experimental blocks on the observed variations. Moreover, 28% of the variability in leaf width was associated with time. The most suitable model for growth analysis was the conditional growth model with a first-order autoregressive moving average structure, homogeneous residual variance-covariance matrix, and a variance-component variance-covariance matrix structure. The analysis of variance for this model revealed significant effects of time and weather conditions on leaf width, while it showed that fertilizers had no significant impact (Table 3).

Concerning leaf length, the model’s null ICC was 0.38%, indicating little block influence on this parameter. Furthermore, time-related variability was estimated at 9.21%, suggesting a low impact of time. The conditional growth model had a homogeneous residual variance-covariance matrix and a variance-component variance-covariance matrix structure. ANOVA on the model indicated significant impacts of time, fertilizers, and weather scenarios (Table 3).

The conditional and marginal explicability coeﬃcients measure the proportion of each growth parameter’s total variance explained by independent factors (weather scenarios, fertilizers, time, and block). These variables contributed low to the total variance of the models developed for crown diameter, leaf length, and leaf width. The block factor had no impact on the total variance for these characteristics. In contrast, independent variables explain 70% of the total height model variance, while the block contributes 10% (Table 3).

**Fig 3:**
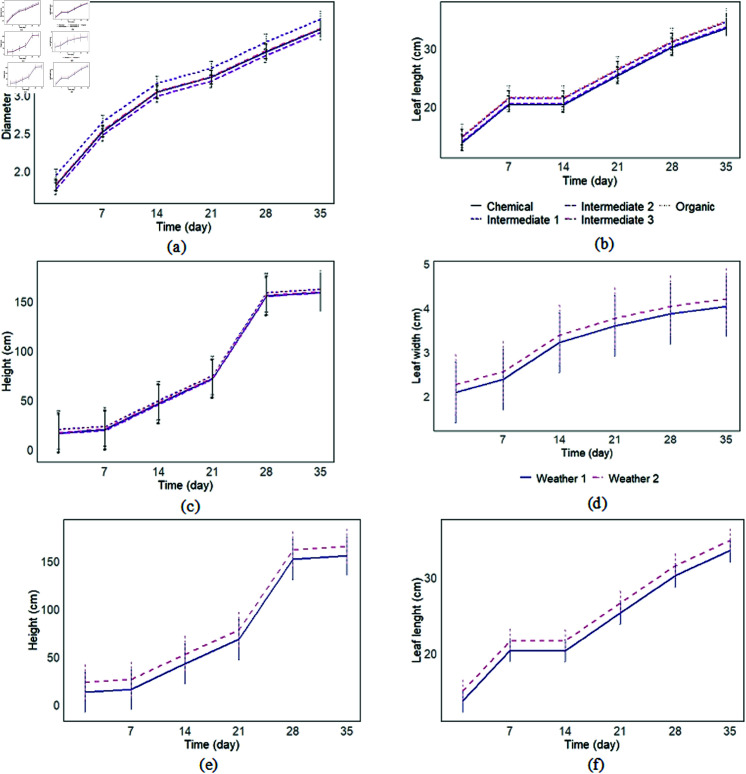
Trend curve illustrates the evolution of each growing trait over time, depending on weather scenarios and fertilizer types: (a) Diameter in cm, (b, f) Leaf lenght in cm, (c, e) Height in cm, (d) Leaf width.

The trends in growth parameters supported the ANOVA findings regarding the models. Maize treated with Intermediate 1 showed a larger diameter than those fertilized with other options. In contrast, Intermediate 2 consistently yielded the most minor diameters throughout the growth period (see Fig 3a). In terms of leaf length, two distinct trends emerged based on the type of fertilizer used: Intermediate 2 and chemical fertilizers exhibited similar trends, while organic fertilizers, along with Intermediate 1 and 3, displayed another set of similarities (see Fig 3b). Additionally, maize fertilized with Intermediate 1 demonstrated greater heights than those treated with other fertilizers between days 1 and 21 and from days 28 to 35 (see Fig 3c). Regarding leaf width, the differences were more pronounced under Weather Scenario 2, which showed a higher growth curve (see Fig 3d), further confirming the results from the ANOVA test. Plants grown in Weather Scenario 2 were also taller than those in Weather Scenario 1 (see Fig 3e). Each factor reached its threshold value on the 28th day. Trends in leaf length varied according to the weather conditions, with higher values noted in Weather Scenario 2 (see Fig 3f).

### Yield parameters

**Fig 4:**
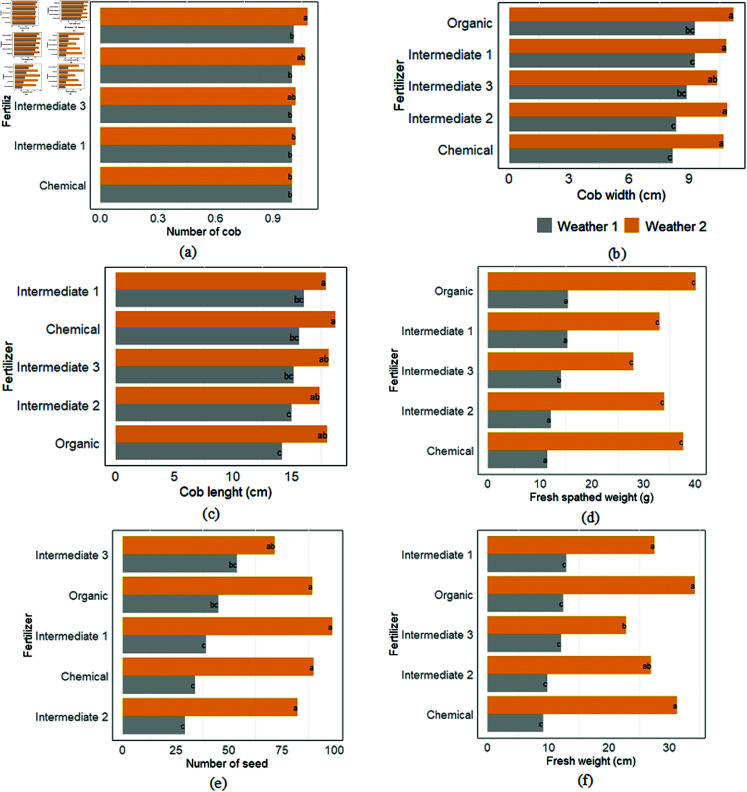
Variation of yield parameters according to fertilizer types and weather scenarios: (a) Number of cob, (b) Cob width in cm, (c) Cob lenght in cm, (d) Fresh spathed weight in g, (e) Number of seed, (f) Fresh weight in g.

The analysis showed that fertilizers significantly influenced the number of cobs, cob dimensions, and fresh maize weight under different weather scenarios. However, no effects were observed for cob width and fresh spathed weight (refer to Fig 4(b), 4(d)). Plants treated with intermediate 2 showed more cobs (Fig 4(a)). Additionally, cobs’ length was mainly observed in weather scenario 2, with high values for intermediate 1 and chemical fertilizers (Fig 4(c)). Fresh maize weights were significantly higher for organic fertilizers (Fig 4(f)). The number of seeds remained high for weather scenario 2 and varied proportionally within fertilizers, except for intermediate 3 (Fig 4(e)). According to the t-test statistics, the yield parameters have a significant effect based on the weather scenarios with the more pronounced impact for Weather scenario 2 (refer to the Supporting information, S3 Table).

Linear mixed-effect models based on cross-sectional data were used to explain yield parameters as a function of block, fertilizers, and weather scenarios. The null model’s intraclass correlation coeﬃcient (ICC) was 1.3e-07%, indicating that the random factor (block) had a negligible impact on the variability observed in the number of cobs. Then, the number of cobs was independent of the block. The best-performing model was the conditional growth model, with a moving-average auto-regressive residual variance-covariance matrix structure. Analysis of variance on this model showed a significant impact of both fertilizer types and weather scenarios on the number of cobs (Table 4).

**Table 4:** Effects of weather scenarios and fertilizer types on maïze grain yield: summary of the linear mixed models. ICC = Intra class correation, DF = Degree of Freedom, F = Fisher Statistics, P = P-value, R^2^m = Marginal Rsquare, R^2^C = Conditional R square.

Yield traits	(Intercept)			Fertilizer			Weather scenarios			ICC (bloc) %	R^2^m	R^2^C
	* DF*	*F/Chisq*	*P*	* DF*	*F/Chisq*	*P*	* DF*	*F/Chisq*	*P*			
Number of cob	-	-	-	4(-)	10.4	0.03	-	11.5	0.0007	1.3 e-07	1.3 e-04	1.3 e-04
Cob lenght	1 (614)	3648.6	<.0001	4 (614)	4.5	0.001	1 (614)	61.7	<.0001	2.09	0.1	0.1
Cob width	1 (614)	53	<.0001	1 (614)	53	<.0001	1 (614)	0.9	0.3	1.5	0.006	0.006
Fresh cob weight in husks	1 (609)	5.1	0.02	4 (609)	1.9	0.1	1 (609)	10.2	0.001	2.9	0.4	0.4
Fresh weight	1 (609)	281.5	<.0001	4 (609)	4.1	0.003	1 (609)	411.1	<.0001	3.6	0.3	0.3
Number of seed	-	-	-	4 (-)	5.2	0.2	1 (-)	40.5	1.91 2e-10	0.1	0.07	0.07

Regarding cob length, the random factor accounted for only 2% of the variability attributed to the block, suggesting a weak impact on this parameter. The optimal model remains the same as for growth parameters. Analysis of variance based on the model shows that fertilizers and weather scenarios affected cob length (Table 4).

The ICC was 1.5% for cob width, showing no impact of block factor was observed. The best model was the conditional growth model with a moving-average auto-regressive residual variance-covariance matrix structure. Following ANOVA, fertilizer types impacted cob width, while weather scenarios had no significant effect on this parameter (Table 4).

The observed variability in maize spathe fresh weight was weakly related to block (2.9%), meaning that fresh maize spathe weight varies little between blocks. Trends between blocks were therefore not presented. The optimal model remains the same as before. ANOVA on this model showed that only the weather scenarios impacted maize spathe weight. The same findings were observed for the number of maize kernels (Table 4).

3.6% of the variability in fresh maize weight was associated with the block. However, this value is less than 5%, so it is not considered significant. The conditional growth model with a moving-average auto-regressive residual variance-covariance matrix structure was identified as the best model. ANOVA on this model showed substantial effects of weather scenarios and fertilizers on fresh weight (Table 4). The models’ estimated marginal and conditional explicability coeﬃcients show that block, fertilizers, or weather scenarios cannot only explain yield parameters. Fresh weight and maize spathe weight had coeﬃcients of 30% and 40%, respectively, indicating that the block did not influence the variances of these parameters. Other factors, such as soil type, edaphic parameters, water availability at different growth phases, and genetic characteristics, can be added to the model to refine the explanation of the various growth parameters in future work.

## Discussion

This research evaluated the impact of weather scenarios and fertilizer types on maize germination, growth and yield parameters. Various analyses were carried out to achieve this goal. Survival analysis models, including Cox, DNNsurv were initially used to model germination time and were compared, while Kaplan-Meier evaluated the probability of germination time. Moreover, linear and generalized mixed-effect models analyzed growth parameters according to time, block, fertilizer types, and weather scenarios. They were also used for yield parameters related to block, fertilizer types, and weather scenarios. Consequently, the DNNsurv model was the most accurate predictor of maize kernels germination time. Additionally, most kernels germinated on day 5, with over 80% of seeds sprouting before day 10. Linear mixed-effect models indicated that weather scenarios, fertilizer types, and time significantly influenced growth parameters, while weather scenarios and fertilizer types affected yield parameters. Furthermore, it was noted that growth and yield parameters were significant for weather scenarios 2, organic, chemical, and intermediate 1 fertilizers.

The research by Hao et al. [39] aligns with the conclusions drawn in this study. Authors reported that the DNNsurv model outperformed other models on high-dimensional survival datasets. Atlam et al. [40] found that the Cox and Deep Learning models performed better when analyzing COVID-19 survival data. The observed DNNsurv performance on maize germination time can be attributed to the Cox model’s reliance on risk proportionality, which assumes that the influence of covariates remains constant over time. However, complex interactions that are better captured by DNNsurv models, which are based on artificial neural networks, affect seed germination. The low concordance index suggests that additional factors, such as soil conditions, water availability, climatic scenarios, and fertilizer type, may better explain the variation in maize germination time. Badr et al. [41] found that the germination time for maize can vary between four to nine days, sparking curiosity about the factors influencing this variability. Meanwhile, Khaeim et al. [42] researched the impact of temperature and water on seed germination and seedling growth. They concluded that the optimal temperature for rapid germination of maize seeds is 20  ° *C*. In their study, 75% of the seeds germinated up to 1 cm within 48 hours of seeding at this temperature. On the other hand, a lower temperature can prolong the germination time. Other factors can also affect germination time, including seed quality, water availability, light conditions, sowing depth, soil temperature, and cultivation practices, which can influence the eﬃciency and speed of the germination process.

Following the results, fertilizer type and weather scenario impacted growth parameters. Variations due to fertilizer types can be attributed to their specific characteristics. Indeed, chemical fertilizers deliver essential nutrients quickly, promoting rapid plant growth. Intermediate fertilizers enhance the decomposition of organic matter, making nutrients available earlier and stimulating growth, especially in height. In contrast, organic fertilizers decompose more slowly, improving water retention and soil structure, which may lead to prolonged growth and increased leaf length. Several research studies have evaluated the effects of different fertilizers on maize growth parameters. The survey by Kaboneka et al. [43] found that using intermediate fertilizer can speed up soil decomposition, making it easier for plants to assimilate more organic matter. However, Kabuya et al. [44] results contradicted the study results. Authors noted that plant diameter remained constant with fertilizer, while plant height showed significant variation. Meanwhile, Wisdom and Ndana [45] found that fertilizers did not substantially affect growth parameters. However, in their study, intermediate 1 and organic fertilizers had the largest diameters and heights, with only leaf width remaining unaffected. Li et al. [46] and Eleduma et al. [47] found that cow dung can stimulate maize development, increasing plant height, diameter, shoot fresh weight, and root fresh weight. Similarly, Washa et al. [48] observed that plant height was associated with the use of cow dung in their study on maize production in Tanzania’s Iringa rural district. Based on the tendency plot after the linear mixed-effect model, most growth parameters were ideal for weather scenario 2. Then, a broader range of weather conditions encourages plant growth by enhancing photosynthesis and enzymatic activity, which are vital for plant development. Additionally, high humidity facilitates stomata opening, allowing for increased gas exchange and the absorption of carbon dioxide necessary for photosynthesis. It also enhances nutrient availability in the soil and maintains optimal hydration for microbial activity.

According to the yield parameters, cow manure, intermediate 1 and chemical fertilizers were more effective than other fertilizers in enhancing maize productivity in scenario 2 while organic and intermediate 1 were more effective in scenario 1. This contrasts Kabuya et al. [44]’s research, which indicates that specific fertilizers have different impacts on maize productivity. The outcome difference could be due to the decomposition state of cow dung used for fertilization. Dried cow manure, used in this research, takes time to break down before being absorbed by the plant. In contrast, decomposed cow manure enhances the availability of nutrients that the plant’s roots can rapidly absorb. However, Jiang et al. [49]’s research on the impact of combined chemical and organic fertilizers on maize productivity does not support these findings. They concluded that larger yields were produced when both fertilizers were used together. The higher yield observed in some studies can depend on factors such as the composition of organic matter nutrients, weather conditions, and the proportion of each fertilizer used. However, this study found that combining 3/4 chemical and 1/4 organic fertilizers produces a similar yield to using only chemical or organic fertilizers. Interestingly, our findings contradict a previous Fan et al. [50]’s study that concluded cow manure is more effective than chemical fertilizers. However, using chemical fertilizers leads to soil deterioration and progressive leaching. The study by Mdlambuzi et al. [51] confirms that applying chemical fertilizer can lead to high yields. But, it is essential to also take into account weather conditions. According to Yin et al. [52], climatic variation accounts for 42% of total maize variability. Therefore, when aiming to maximize crop yield, it is crucial to consider both the fertilizer types and weather scenarios.

In Benin, maize yield was optimal for both weather associations [22]. Despite both rules being optimal maize growth conditions, weather scenario 2 gave a more crucial yield, with slightly higher values for minimum and maximum temperature and maximum humidity than weather 1. Gyamerah et al. [53] support these results, showing that high temperatures and rainfall positively impacted maize yields in Ghana. The critical range of climatic conditions defined by these authors to maximize maize yield in Ghana was estimated between 27.9 and 28.1  ° *C*. In contrast, the maximum temperature in this study was between 29.4 and 35.9  ° *C*. It should be noted that the association of weather parameters with this temperature also has a high humidity interval, which can benefit plant development and production. Higher relative humidity can reduce plant water stress, promote better nutrient assimilation, and stimulate growth, contributing to higher yields. With Lizaso et al. [54] study on the impact of high temperatures on maize phenology and yield components, warm temperatures accelerate maize development rates, resulting in shorter vegetative and reproductive phases. The research by Bonea and Urechean [55] on the response of maize yield to variations in precipitation and average temperature in the central part of Oltenie demonstrates a significant effect of climate on yield. The authors concluded a positive correlation with rainfall and a negative correlation with temperature. Similarly, Shammi and Meng [56] study on modeling the impact of climate changes on crop yield in Mississippi concluded that a one-degree increase in maximum temperature reduces yield by 2 to 7%. Furthermore, research by Stuch et al. [57] on climate change’s impact on main small-scale crops in main small-scale crop yield variability in sub-Saharan Africa shows a reduction in maize yields of 85% in West Africa, 29% in Central Africa, and 32% in East Africa. In Mali, June and July have average temperatures of 33.16°C and 58.77 mm, respectively, making them unsuitable for maize cultivation in the short and long term [58], while in Benin, both months are thought to be the wettest.

Other climatic parameters besides temperature, relative humidity, and rainfall influence crop production. According to Zhou et al. [59], temperature and radiation during grain filling significantly affect growth rate, filling duration, kernel weight, and maize yield. This aﬃrmation is aligned with Miassi et al. [60]. Future studies may explore the complex interactions between weather parameters and other factors influencing crop production to better understand and anticipate crop productivity dynamics.

## Conclusion

This study evaluated the effects of several combinations of weather and fertilizer parameters on maize germination time, growth, and yield characteristics. To this purpose, maize germination time was modeled using Cox and Deepsurv survival models. Likewise, growth and yield parameters were subjected to both linear and generalized mixed-effects models. Consequently, most of the kernels germinated on day four following sowing. However, germination time was independent of the type of fertilizer and weather scenarios. Furthermore, the autoregressive growth model best fits all the maize growth and yield parameters. The fertilizer types and weather scenarios significantly affected growth and yield parameters, except for the cob diameter and width leaf for the weather scenario and the cob number for the fertilizer type. On the other hand, yield parameters were twice as high under weather scenario 2. Regarding maize fresh weight, organic, chemical, and intermediate 1 fertilizers performed statistically similarly and better than other fertilizers. However, yield remains statistically identical between the fertilizers applied under weather scenario 1. These findings highlight the importance of considering how farming practices interact with climate change to improve yields. Additionally, the comparable effectiveness of organic, chemical, and intermediate fertilizers in scenario 2 suggests that adapting these methods could increase crop resilience. These findings can help identify effective agricultural practices to sustain productivity in the face of climate change.

## Supporting information

S1 Fig Experimental design. (TIF)

S1 Fig Kernel emergence according to treatments with the Kaplan-Meier model. This figure shows that maize emergence time does not vary according to fertilizer type or climatic association. (TIF)

S1 Table Models performance based on Rsquare: Coeﬃcient of determination, RMSE: Root mean square error, MA: mean absolute error. (PDF)

S1 Table T-test comparing the individual effects of growth parameters across whether associations. (PDF)

S3 Table T-test comparing the individual effects of yield parameters across whether associations. (PDF)
