# Supplementary figures and images for: Effects of weather scenarios and fertilizer on maize growth and yield: Insights from a greenhouse experiment
Source: PLoS One. 2025 Mar 3;20(3):e0318121. doi: 10.1371/journal.pone.0318121 (PMC11875340; doi:10.1371/journal.pone.0318121)

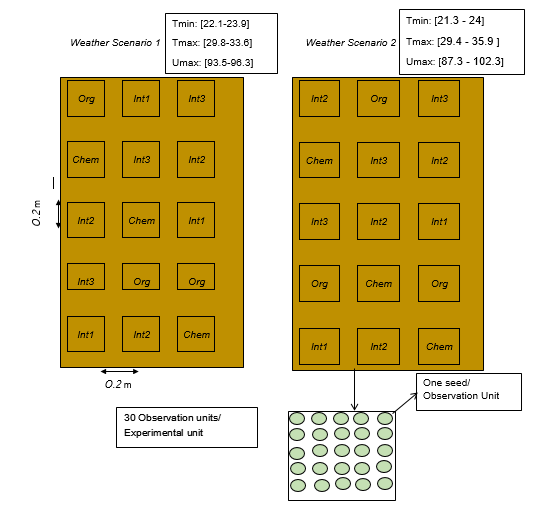

Supplement: S1 Fig — (TIF) [file pone.0318121.s001.tif]

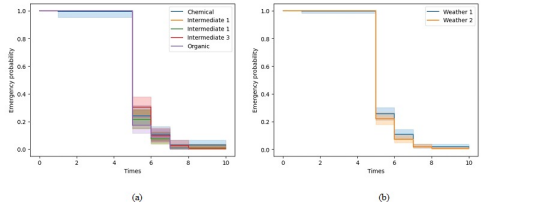

Supplement: S1 Fig — (TIF) [file pone.0318121.s002.tif]
