# Supplementary material for: Effects of weather scenarios and fertilizer on maize growth and yield: Insights from a greenhouse experiment
Source: PLoS One. 2025 Mar 3;20(3):e0318121. doi: 10.1371/journal.pone.0318121 (PMC11875340; doi:10.1371/journal.pone.0318121)
Supplement: S1 Table — (PDF) [file pone.0318121.s003.pdf]

S1 Table: T-test comparing the individual effects of growth parameters across whether associations

Test parameters	Diameter	Height	Number of leaves	Leaf lengh	Leaf Width
			Organic		
t	-5.35	-5.4	-0.7	-4	-3.02
p	0	0	0.49	0	0
			Chemical		
t	1.31	-8.16	4.18	-2.11	-0.25
p	0.19	0	0	0.04	0.8
			Intermediate 3		
t	2.77	-6.84	2.47	-2.71	-2.78
p	0.01	0	0.01	0.01	0.01
			Intermadiate 2		
t	-3.22	-8.17	4.63	-3.76	-2.66
p	0	0	0	0	0.01
			Intermadiate 1		
t	1.01	-7.7	3.14	-3.56	-4.93
p	0.32	0	0	0	0

S2 Table shows the results of a t-test designed to evaluate the effects of various growth parameters on different climatic associations. It reveals a significant difference in plant diameter for organic, intermediate 2, and 3 fertilizers. In terms of leaf length, considerable variations were observed according to climate. The number of leaves remains similar for both climatic associations when plants received organic fertilizers, while leaf width was constant when exposed to the same type of organic fertilizer.
