# Supplementary material for: Effects of weather scenarios and fertilizer on maize growth and yield: Insights from a greenhouse experiment
Source: PLoS One. 2025 Mar 3;20(3):e0318121. doi: 10.1371/journal.pone.0318121 (PMC11875340; doi:10.1371/journal.pone.0318121)
Supplement: S1 Table — (PDF) [file pone.0318121.s004.pdf]

S2 Table: T-test comparing the individual effects of yield parameters across whether associations

	Number cob	Cobs length	Cob width	Number seed	Fresh weight cob leaf	Fresh weight cob
				Organic		
t	-1.79	-3.79	-5.17	-3.43	-5.65	-5.5
p	0,08	0	0	0	0	0
				Chemical		
t	-	-3.86	-6.93	-4.03	-8.35	-7.09
p	-	0	0	0	0	0
			Intermediate 3			
t		3	-3.47	-1.63	-3.6	-3.02
p		0	0	0,11	0	0
			Intermediate 2			
t		3.37	-5.95	-5.16	-7.97	-6.74
p		0	0	0	0	0
			Intermediate 1			
t		3.66	-6.62	-5.64	-8.72	-7.61
p		0	0	0	0	0
