# Supplementary material for: Effects of weather scenarios and fertilizer on maize growth and yield: Insights from a greenhouse experiment
Source: PLoS One. 2025 Mar 3;20(3):e0318121. doi: 10.1371/journal.pone.0318121 (PMC11875340; doi:10.1371/journal.pone.0318121)
Supplement: S3 Table — (PDF) [file pone.0318121.s005.pdf]

S3 Table: Models performance based on Rsquare: Coefficient of determination, RMSE: Root mean square error, MA: mean absolute error

	DT	RF	XGBoost	Adaboost	GB	BR	Light GBM
	Diameter						
RMSE	0.58	0.56	0.55	0.58	0.56	0.58	0.57
Rsquare	0.59	0.60	0.61	0.57	0.60	0.56	0.60
MAE	0.45	0.43	0.42	0.44	0.43	0.45	0.44
				Height			
RMSE	19.18	19.6	19.24	19.7	19.7	23.14	21.82
Rsquare	0.88	0.88	0.88	0.87	0.88	0.76	0.88
MAE	11.70	11.70	11.7	12.27	11.69	16.50	14.9
				Lenght leaf			
RMSE	4.79	4.89	4.94	4.97	4.87	5.01	4.97
Rsquare	0.45	0.43	0.42	0.41	0.44	0.37	0.44
MAE	3.69	3.77	3.84	3.87	3.75	3.83	3.81
				Wide leaf			
RMSE	0.54	0.53	0.66	0.65	0.46	2.65	1.95
Rsquare	0.66	0.66	0.54	0.65	0.53	0.65	0.66
MAE	0.42	0.42	0.42	0.43	0.42	2.05	1.50
